# Anti-amyloid-β Monoclonal Antibodies as Promising Disease-Modifying Therapies in Alzheimer’s Disease: A Focus on Aducanumab, Lecanemab, Crenezumab, Gantenerumab and Solanezumab

**DOI:** 10.1192/j.eurpsy.2023.663

**Published:** 2023-07-19

**Authors:** V. S. D. Melo, C. A. Rodrigues, I. A. Silva

**Affiliations:** ^1^Psychiatry, Centro Hospitalar do Médio Tejo, Tomar; ^2^Psychiatry, Unidade Local de Saúde do Norte Alentejano, Portalegre, Portugal

## Abstract

**Introduction:**

Alzheimer’s disease (AD) is the most prevalent form of age-related dementia in the world. The body of evidence suggesting that its main pathological features consist of amyloid-β (Aβ) plaque deposits and neurofibrillary tangles formed by hyperphosphorylated tau protein is robust. The drugs currently on the market have no effect on disease progression and provide only partial symptomatic relief, which creates a large unmet medical need. Anti-Aβ monoclonal antibodies (mAbs) have been shown to reduce amyloid plaques. Therefore, passive immunization is a major hope for treatment of AD.

**Objectives:**

This review aims to summarize the up to date knowledge and experience with Anti-Aβ mAbs with positive clinical or biomarker effects in long-duration trials.

**Methods:**

A narrative review was conducted based on a search in *Google Scholar* and *Pubmed*, using the following terms or combinations “anti-aβ protofibril antibody”; “early alzheimer’s disease”; “immunotherapy for Alzheimer’s disease”. Peer-reviewed literature published between 2016 and April 2022 was screened on full-text for this purpose.

**Results:**

Aducanumab surpassed a successful Phase 1B trial demonstrating a dose and time dependency for Aß reduction with a beneficial impact on some clinical measures after 1 year of treatment. Two large Phase 3 clinical trials were initiated and already discontinued based on futility analysis done and not based on safety concerns. Further analyses including participants exposed for longer periods of time at higher doses indicated that aducanumab reduced brain amyloid and decreased the rate of decline.

Lecanemab (BAN2401) completed a Phase 2 trial (2018) with evidence of amyloid reduction and slowing of cognitive decline and has now entered Phase 3. Aducanumab and BAN2401 showed significant efficacy on both clinical and biomarker outcomes.

Crenezumab Phase 2 trial results suggested efficacy in mild AD; a Phase 3 program was recently halted due to futility. This mAb is currently being assessed in a prevention trial involving a Colombian kindred with autosomal dominant AD.

Gantenerumab showed significant biomarker effects, with no clinical efficacy reported to date and is being assessed in Phase 3 trials after a trial in prodromal disease stopped for futility suggested that higher doses might be efficacious. Gantenerumab and solanezumab showed no drug-placebo differences in clinical outcomes of specific studies included in this review.

**Conclusions:**

Therapies with anti-Aβ mAbs have been developed successively and conducted in clinical trials signaling a promising new era for AD drug development and providing compelling evidence for the prominent role of neurotoxic soluble amyloid oligomers in the pathogenesis of AD and as therapeutic targets. Lessons learned from these studies may also be a bridge to more efficacious, safe drugs in AD.

**Disclosure of Interest:**

None Declared

